# Potential role of exosome in post-stroke reorganization and/or neurodegeneration

**DOI:** 10.17179/excli2020-3025

**Published:** 2020-12-11

**Authors:** Fateme Azizi, Sahar Askari, Pegah Javadpour, Mahmoudreza Hadjighassem, Rasoul Ghasemi

**Affiliations:** 1 Department of Neuroscience and Addiction Studies, School of Advanced Technologies in Medicine, Tehran University of Medical Sciences, Tehran, Iran; 2 Department of Physiology, School of Medicine, Shahid Beheshti University of Medical Sciences, Tehran, Iran; 3 Brain and Spinal Cord Injury Research Center, Neuroscience Institute, Tehran University of Medical Sciences, Tehran, Iran; 4 Neurophysiology Research Center, Shahid Beheshti University of Medical Sciences, Tehran, Iran

**Keywords:** stroke, neurodegenerative diseases, exosome, miR9, miR124

## Abstract

Currently, stroke is a common and devastating condition, which is sometimes associated with permanent cerebral damages. Although in early time after stroke, the related treatments are mainly focused on the restoration of cerebral blood flow (CBF), at the same time, some changes are commencing that continue for a long time and need to be specially noticed. Previous studies have proposed several molecular mechanisms in these post-stroke events. Exosomes are a type of vesicle, which are formed and secreted by most cells as a mean to transfer cellular constituents such as proteins, DNA and/or RNA to distant cells. Therefore, they are considered as a novel mechanism of cellular communication. Herein, we reviewed the current knowledge on cascades, which are activated after stroke and consequently lead to the reorganization and/or continuance of tissue damage and development of other disorders such as Neurodegenerative diseases (ND). Thereafter, we summarized the latest proofs about the possible participation of exosomes in transferring some components such as proteins and micro-RNAs (miRs), from the affected areas to other parts of the brain and eventually cause the above-mentioned post-stroke events.

## Introduction

Stroke is known as an important cause of death worldwide and its long-term complications devastate the affected patients and their families (Maaijwee, 2014[81]). Although performing timely intervention to save patients' life appears to be important, in recent years, an increasing attention has been paid to modulate the stroke's subsequent consequences and improve the quality of life in these patients (Linder et al., 2015[73]). As a result of brain reorganization, which starts after a stroke, the limited spontaneous recovery of function occurs. On the other hand, the injury may negatively affect other brain's areas, which is a process that causes other disorders (Zhang and Chopp, 2016[135]). In the same way; there are studies indicating a close link between stroke and the increased risk of developing Neurodegenerative diseases (ND), which might occur a few years after stroke (Mijajlović et al., 2017[83]). To date, there is limited evidence about the molecular mechanisms underlying the post stroke reorganization and/or degeneration. Accordingly, exosomes are among those, which were shown to contribute in both processes. Moreover, exosomes have been recognized as the important mediators of long-distance intercellular communication. They have a well-defined lipid bilayer with surface markers that carry a variety of biological materials including proteins and nucleic acids (Zhang et al., 2019[134]).

Given that, the current knowledge about exosomes is expanding and there are clear proofs about their contribution in reorganization and/or degeneration. Therefore, herein, we highlighted recent insights into the role of exosomes in brain reorganization processes and/or degeneration after stroke.

## Stroke

Stroke, as the third leading cause of death worldwide, is a brain damage that occurs when the blood supply to the brain ceases and subsequently the brain function is rapidly lost (Chugh, 2019[28]). Thereafter, the sudden death of brain cells occurs due to ischemia (loss of blood supply) or hemorrhage (rupture of a blood vessel that supplies the brain). Less commonly, a stroke is caused by a lack of blood flow to the entire brain area due to cardiac arrest (Lo et al., 2003[76]). 

The elderly are mostly affected by stroke, however, its frequency in the young population is also increasing. The occurrence of stroke among young people causes disability during their life productive years, which imposes an economic burden on the individual and the society (Smajlović, 2015[106]). Furthermore, this disease initiates a pathophysiological cascade that leads to irreversible death of neuronal cells as well as neurological dysfunction. In this regard, the symptoms depend on the affected area of the brain and neurological deficits also reflect the location and size of this brain area affected (Lo et al., 2003[76]).

Thus, the primary goal for the treatment of ischemic stroke is rapid restoration of cerebral blood flow (CBF) before passing the critical time point. However, re-establishment of CBF and minimizing damage cannot salvage irreversibly damaged brain cells, which ultimately leads to long-term disability in people who survived (Moskowitz et al., 2010[85]). It is noteworthy that the most notable stroke disabilities commence in a short time, but some of them occur even a few years later.

Following stroke, a series of changes initiate, some of which are functionally beneficial and others may result in neurodegeneration. Accordingly, one of these changes is the reorganization of the connectivity patterns of surviving neurons, which takes place to support the lost functions (Jones and Adkins, 2015[59]). In addition, another form of these changes is the induction of neurodegeneration in the brain, which may occur later (Cumming and Brodtmann, 2011[30]). In the succeeding sections, these changes and factors, which may play roles in these events, are discussed.

### Post-stroke brain reorganization 

**Figure 1:**
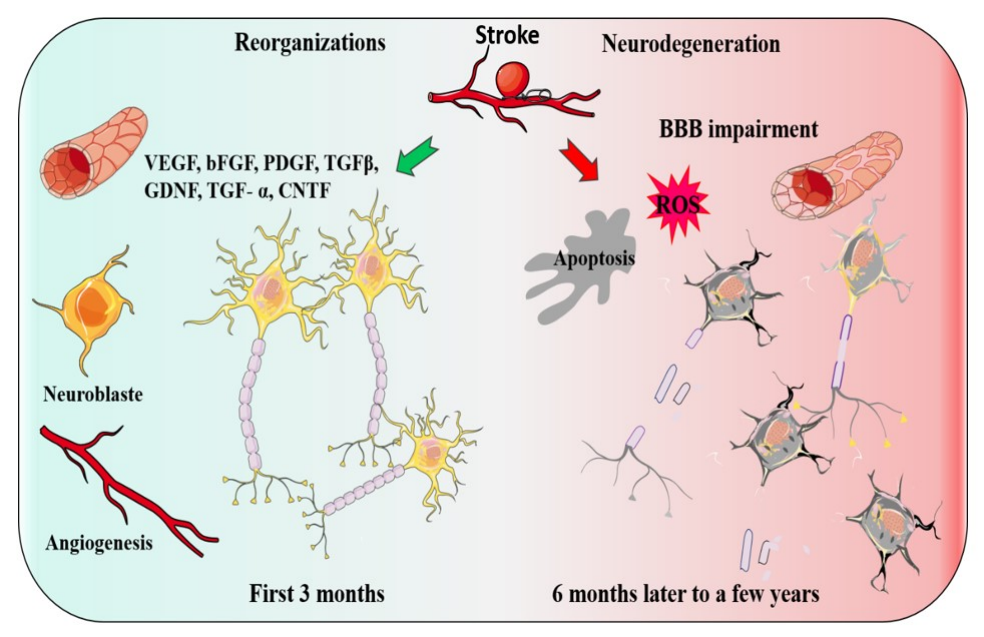
Two events may occur after a stroke, Reorganization or Neurodegeneration. The green arrow indicates the organization and the factors involved. Two coupled processes, angiogenesis and neurogenesis and growth factors play a key role in a functional reorganization after stroke. The red arrow indicates the factors which may lead to post-stroke Neurodegeneration.

After a stroke, several functions that were previously performed by the ischemic region would be performed by other ipsilateral or contralateral brain regions, which is a process named as brain reorganization (Green, 2003[48]). Recently, novel insight into cellular and molecular mechanisms has been gained underlying brain reorganization processes. Brain reorganization events are mediated by intracellular and intercellular molecules that exert their effects through autocrine and paracrine signaling (Zhang and Chopp, 2016[133]). Evidence suggests that two coupled processes, namely angiogenesis and neurogenesis play key roles in a functional reorganization after stroke (Ohab et al., 2006[89]) (Figure 1(Fig. 1)).

Angiogenesis is a physiological process, through which new blood vessels are generated from pre-existing vascular Epithelial cells (ECs), to supply nutrients and oxygen and to promote stroke recovery (Carmeliet, 2005[22]). Notably, the proliferation of cerebral ECs is strictly controlled by many key angiogenic factors in the central nervous system (CNS) of the adult brain. One of the situations, which is shown to induce angiogenesis in adult human and rodent brains, is the response to various pathological conditions. However, angiogenesis is not only restricted to stroke, but it is also induced in other situations, and the magnitude of this process is more pronounced following stroke (Yin et al., 2015[129]). In patients who experienced stroke, the number of new vessels showed a notable correlation with longer survival times, which suggests that an active angiogenesis may be beneficial (Krupinski et al., 1994[64]). 

As noted earlier, neurogenesis is another element of reorganizations after stroke. In pathological conditions such as stroke, the brain tries to repair itself by producing new neurons in those areas where less neurogenesis is seen to occur normally (Lu et al., 2017[78]). Stroke-induced compensatory neurogenesis is shown to contribute to the post-ischemic recovery seen in both human and animal's brains (Han et al., 2019[51]; Ohab et al., 2006[89]). In this regard, several studies indicated that stroke induces alterations of the neural stem cell and the vascular architecture of the adult sub-ventricular zone (SVZ) neurogenic niche, by passing 3 months from the onset of stroke in experimental animals (Zhang et al., 2014[133]). 

Thus, neural reorganization after stroke is thought to be an intrinsic capacity of the brain to compensate for structural damage through the reorganization of surviving networks. Apart from these effects, stroke can also initiate some other effects, which are not as favorable to previous ones, named as commencing a cascade of neurodegeneration in different loci of the brain.

### Post-stroke neurodegeneration

According to previous studies, it has been widely accepted that there is a close link between stroke and the increased risk of developing dementia by passing a few years from stroke (Allan et al., 2011[4]; Altieri et al., 2004[5]; Sachdev et al., 2014[97]; Tatemichi et al., 1990[114]). In this regard, a study has revealed that 4 and 7 days after the induction of focal cerebral ischemia in rats, accumulation of β-amyloid was evident at the periphery of the infarcted area (Stephenson et al., 1992[109]). In addition, there is evidence from larger species animals stating that they have a longer natural life span than rodents. In 1990, Uchida et al. in their study found that amyloid angiopathy is associated with cerebral hemorrhage and senile plaques in the brain of aged dogs (Uchida et al., 1990[119]). Besides animal studies, human evidences for this link were also provided by cross-sectional (Barba et al., 2000[11]; Desmond et al., 2000[34]; Pohjasvaara et al., 1997[92]), Cohort epidemiological (Desmond et al., 2002[35]; Tatemichi et al., 1990[113]) and post mortem studies (Schneider et al., 2004[102]).

Although stroke mainly occurs in older adults; and therefore, these patients may have had a cognitive decline before stroke (Henon et al., 1997[52]), a recent study has shown that the prevalence of stroke in the young population is increasing and these patients usually confront higher risks of post-stroke dementia compared to older patients. This risk is increased ≈2-fold compared with the general population and then remains persistently high even after 10 years (Corraini et al., 2017[29]). Likewise, various studies have confirmed that ischemic brain injury consequently results in a complex sequence of pathophysiological events that involve region and time-dependent disturbances. Such delayed progression in brain damage might lead to neurological deficits over time in patients who have survived ischemic stroke (Dirnagl et al., 1999[37]; Mijajlović et al., 2017[83]). 

In the following sections, some of the mechanisms that might be involved in the incident post-stroke reorganization and/or degeneration, are summarized.

### Reorganization promoting factors

There are some major factors identified to be associated with the development of post-stroke reorganizations. Vascular endothelial growth factor (VEGF), Basic fibroblast growth factor (bFGF), platelet-derived growth factor (PDGF) and transforming growth factor-beta (TGFβ), epidermal growth factor (EGF), BDNF, bone morphogenetic protein (BMP), glial cell-derived neurotrophic factor (GDNF), transforming growth factor- (TGF-) α, and ciliary neurotrophic factor (CNTF), are some examples, which have been proposed to play essential roles in the adult reorganizations response to ischemia stroke (Chou et al., 2006[27]; Kitagawa et al., 1999[61]; Kokaia et al., 1995[63]; Lin et al., 1997[72]; Sato et al., 2016[100]; Tsai et al., 2006[116]; Türeyen et al., 2005[118]; Yin et al., 2015[129]) (Figure 1(Fig. 1)).

In addition, accumulated evidence is available in this regard, which suggests that several microRNAs (miRs) by regulating target genes at the post-transcriptional levels, are involved in the reorganization process after stroke (Liu et al., 2013[75]; Stappert et al., 2018[108]). MiRs family has been discovered as a novel family of non-coding small RNAs that play important gene-regulatory roles and modulate protein expression in various organisms (Bartel, 2009[14]). Moreover, compelling evidence exists supporting the changes in the expression pattern of miRs following reperfusion in stroke (Jeyaseelan et al., 2008[57]). Likewise, an increasing number of investigations have examined the roles of specific miRs in angiogenesis (Li et al., 2015[71]; Shi et al., 2018[104]; Zhao et al., 2018[136]) and neurogenesis processes following a stroke (Åkerblom et al., 2012[2]; Cheng et al., 2009[25]). In this regard, several studies have shown that some miR are strongly expressed in the ischemic boundary zone after stroke, which regulate post-stroke angiogenesis by increasing the expression of angiogenesis factors as well as promoting proliferation, migration, and angiogenesis abilities of ECs. Therefore, miRs may serve as a novel therapeutic tool in stroke treatment (Li et al., 2015[71]; Shi et al., 2018[104]; Su et al., 2017[110]). In addition, stroke induces neurogenesis including proliferation and differentiation of neural progenitor cells as well as the migration of newly generated neuroblasts. Emerging data indicated that stroke alters miRNA expression in SVZ neural progenitor cells and miRNAs also play roles in mediating proliferation and differentiation processes of adult neural progenitor cells (Liu et al., 2011[74], 2013[75]). On the other hand, functional magnetic resonance imaging (fMRI) and Positron emission tomography (PET) in human stroke victims clearly demonstrated that both hemispheres are involved in the recovery after stroke (Carey et al., 2002[21]; Chollet et al., 1991[26]; Guan et al., 2011[49]). 

Therefore, stroke has a broad effect on angiogenesis and neurogenesis that not only affect peri-infarct regions, but also affect the areas that are far from the site of injury. Cell-to-cell communication plays a key role in the dynamic modulation of angiogenesis and neurogenesis in both hemispheres after stroke. Given that these distant areas should communicate with each other in above-mentioned situations, currently the question is that how extracellular signals originated from infarcted area reach the distant locations, including the other hemispheres. It seems that the means, by which cargo is transmitted, should prevent cargo from being destroyed by enzymes and should deliver them intact to the target cells. Compelling evidence has shown that exosomes because of its features, are considered as possible candidates in transmitting post-stroke reorganizations signaling from the damaged hemispheres to distant compensatory areas.

### Neurodegeneration promoting factors

The most common ND are amyloidopathies, tauopathies, and synucleinopathies, which are represented by AD and PD (Dugger and Dickson, 2017[39]). Accordingly, stroke is a well-known risk factor for dementia. However, numerous aspects of this association, which could help to prevent dementia effectively, are poorly understood yet. In recent years, researchers have been attempting to understand the linkage seen between these two disorders (Corraini et al., 2017[29]). In the following sections, the possible mechanisms attributed to the increased rates of ND after stroke are discussed.

A large cohort study by including more than 1000 patients with stroke, showed that, in addition to age and number of infarcts, chronic changes in white matter also are important risk factors for dementia after stroke. Correspondingly, these chronic changes in the brain are likely to endanger cognitive reserves and increase the risk of dementia after a stroke (Yang et al., 2015[126]). 

In this regard, a previous study investigated the impact of the single nucleotide polymorphism (SNP) in endothelial nitric oxide synthase (NOS3) gene on the development of pathological substrates and incident dementia. As a result, they reported that NOS3 is potentially associated with incident dementia in elderly stroke survivors, which may be mediated by the reduced nitric oxide production and cerebral perfusion (Morris et al., 2011[84]). In addition, some studies that investigated SNP showed that it is in the gene encoding the angiotensin-converting enzyme, as an intriguing genetic risk factor for the development of dementia following a stroke. However, these articles obtained controversial findings (Arpa et al., 2003[7]; Bour et al., 2010[18]; Chapman et al., 1998[24]; Packard et al., 2007[91]; Slooter et al., 1997[105]). 

The structural and functional integrities of blood vessels and adequate blood supply are the key factors for the normal functioning of the brain (Sweeney et al., 2018[111]). Recently, a report has been published suggesting that chronic cerebral hypoperfusion triggered by stroke may subsequently disturb Aβ clearance, and this could be recognized as one of the mechanisms that may contribute to the development of post-stroke dementia (Back et al., 2017[8]). Furthermore, blood brain barrier (BBB) impairment is another common finding in ND and stroke (Yang and Rosenberg, 2011[128]). In this regard, a recent study revealed that in a mice model of transient middle cerebral artery occlusion, BBB exhibits biphasic openings in both early and late time points after ischemia (by passing 6 and 72 hours, respectively) (Hone, 2018[54]). Although further studies are needed to investigate BBB integrity for a longer-time, given this observation, it is logical to assume that post-stroke CBF shortfalls and BBB impairment are involved in developing ND by passing a few years from stroke. In addition, inflammatory changes in the brain after ischemia could be associated with post-stroke cognitive decline. In this regard, a number of studies have shown that erythrocyte sedimentation rate (ESR), serum levels of C-reactive protein (CRP), interleukins are associated with subsequent poor cognitive performances following stroke (Kliper et al., 2013[62]; Narasimhalu et al., 2015[88]; Rothenburg et al., 2010[96]).

Some studies indicated that, abnormal proteins in ND may transfer from cell to cell along with anatomically connected pathways, which may explain some of the specific anatomical patterns of cell death (Brettschneider et al., 2015[19]). For instance, alpha-synuclein, which is a protein that its abnormal aggregation contributes to PD pathology, spreads to other neurons by various mechanisms such as tunneling nanotubes, classical exocytosis and endocytosis, trans-synaptic junctions, and direct penetration (Xia et al., 2019[124]). On the other hand, abnormal proteins in ND were previously known as intracellular proteins; however, recent studies have established that they can be detected in the plasma and CSF of human beings as well as in the culture media of neuronal cells (Emmanouilidou et al., 2010[40]). Furthermore, several different miRNAs and their target genes were recognized to be involved in the pathophysiology of neurodegenerative and ischemic stroke (Eyileten et al., 2021[41]). 

Given the above-mentioned results, it is conceivable to assume that safe transmission of these protein cargoes to distant neurons could be a missing ring in the relationship between post-stroke events and the development of ND. In recent years, studies indicated that this process could be mediated through a newly defined mechanism, named as exosomes, (D'anca et al., 2019[32]) which are discussed in more details below.

## Exosome

### History of the exosome

142 years ago, Edmunds observed some particles in normal serum with the dark-ground illumination. Since the functions of these particles were unclear and the main mass of these particles was proved to be fat, Muller [1896] called these particles as blood dusts (Frazer and Stewart, 1937[44]). In 1962, Barland saw a clearer structure of cellular vesicles in a microscope (Barland et al., 1962[12]). 

Thereafter, in 1981, an examination performed using electron microscopy showed the extracellular vesicles with an average diameter of 500 to 1000 nm and the term exosome was firstly used for describing these particles (Trams et al., 1981[115]). Since then, some studies were conducted on exosomal characteristics and functions that extended quickly. However, the function of the exosomes still remained largely unknown.

### Physiology of exosome

**Figure 2:**
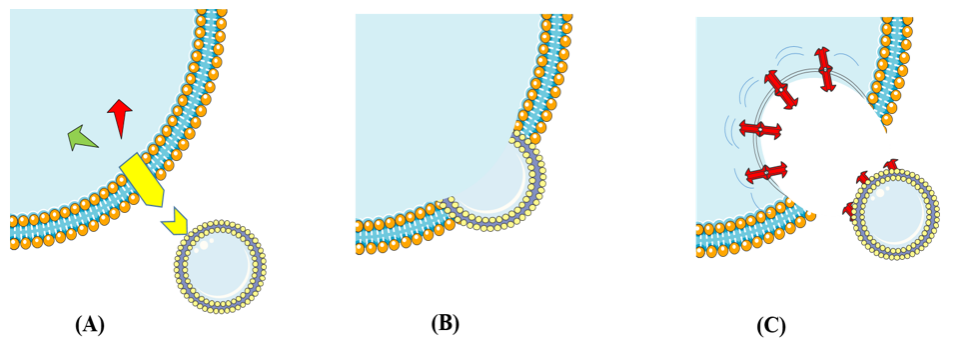
This schematic representation illustrates cell-to-cell communication through exosomes. (A) Exosomes attach to the cell surface and bind to receptors (Yellow icon) to activate intracellular signaling pathways. (B) Exosomes fusion with the target cell membrane. (C) Exosomes enter target cells by endocytic mechanisms (phagocytosis, macropinocytosis, or receptor-mediated endocytosis).

Exosomes are bilayered lipid membrane-enclosed vesicles (30 to 100 nm in diameter), which are produced and released by most cell types, but not all of them (Record et al., 2011[94]). Additionally, they contain various biological materials including proteins, lipids, and genetic materials, which are involved in intercellular communication between source and target cells under physiological and pathophysiological conditions. The exosomal cargo is packaged and then enclosed inside the exosome membrane, which consequently prevents their destruction by enzymes and delivers them in intact form to targeted cells. However, it is still unclear whether exosomes are exclusively “addressed” to target cells (Brinton et al., 2015[20]), but it has been shown that exosomes have interactions with their target cells in different ways (Figure 2(Fig. 2)).

In the first model, they can attach to the cell surface and then bind to receptors, in order to activate intracellular signaling pathways without any internalization. In another way, they fuse with the target cell membrane and then obtain new receptors, enzymes or genetic material from the vesicles, which gives new properties to target cell. In the last but not the least model, the interaction between exosome and cell membrane provides a pathway used by target cells to internalize exosomes by endocytic mechanisms (phagocytosis, macropinocytosis, or receptor-mediated endocytosis) (Fitzner et al., 2011[43]; Mulcahy et al., 2014[86]). 

Exosomes are enriched with parent cell-derived surface markers as follows: TSG101, Alix, flotillin 1, tetraspanins (CD9, CD63, CD81), integrins, and cell adhesion molecules (CAM) (Akers et al., 2013[3]; Batrakova and Kim, 2015[16]; Lötvall et al., 2014[77]). Exosomes can also be isolated from the conditioned cell culture media or bodily fluids, but samples of biofluids contain a combination of exosomes with different cellular origins. To explore exosomes from specific tissues or cells, the best way is to obtain them from the medium of the cultured cells (Batrakova and Kim, 2015[16]; Tschuschke et al., 2020[117]). 

Up to now, diverse methods of exosome isolation have been proposed and developed. Correspondingly, each method or approach has its own advantages and disadvantages that can separate exosome with different purities and qualities. Different techniques for the separation of exosomes are used including ultracentrifugation, ultrafiltration, size exclusion chromatography (SEC), polymer precipitation, immunoaffinity chromatography, and techniques based on microfluidics. Notably, reviewing the methods of sample preparation and exosome isolation is beyond the scope of this review article, so they are reviewed elsewhere (Doyle and Wang, 2019[38]; Li et al., 2017[70]; Tschuschke et al., 2020[117]).

### Exosome in Central Nervous System

Neurons are the brain cells responsible for the rapid communication of information. Inter-neuronal communications in the CNS is divided into two categories. The first one is the classical form of cellular communication, named as wiring transmission (point-to-point communication), which is mediated by neurotransmitters and the second one is volume transmission (VT) (communication in the extracellular and biological fluid) that involves a large number of cells in the CNS. It is noteworthy that there are different forms of VT. According to some previous studies, VT is mediated by diffusion and flow of soluble biological signals such as transmitters and modulators. However, emerging data indicated that exosome represent a novel type of VT (Agnati and Fuxe, 2014[1]; Borroto-Escuela et al., 2015[17]). Most cell types in CNS including neurons and glial cells, are capable of releasing exosomes. Notably, neurons have a complex morphology; therefore, they have more expanded trafficking and signaling needs compared to those of geometrically simpler cells. In neurons, multivesicular bodies, which contain exosomes, are predominantly found in cell bodies and dendrites of both peripheral nervous system (PNS) and CNS. Correspondingly, they are about 50 times more abundant in the soma or dendrites than in axons (Lafourcade et al., 2016[66]). Recently, studies have established a novel concept stating that exosomes and their cargo play roles in anterograde and retrograde signaling across synapses and then represent a novel way for the intercellular exchange of proteins and RNAs within neural networks (Lachenal et al., 2011[65]). 

A growing body of evidence suggests that exosomes are involved in several processes needed for normal brain function and neural support such as intercellular communication, the myelin sheath maintenance, and waste elimination. Furthermore, they have a functional effect on nerve development, activation, regeneration, neurogenesis, plasticity, and neuroinflammation (Bátiz et al., 2016[15]; Gómez-Molina et al., 2019[47]; Luarte et al., 2017[79]). 

Altogether, these findings suggest that exosomes play important roles in neuronal communications in the CNS.

## The Role of Exosomes in Stroke and Post-Stroke Events

### The role of exosomes in stroke and brain reorganization

In addition to their critical role in normal brain function as well as in nerve regeneration, plasticity, immune response, and synaptic function (Bahrini et al., 2015[9]; Li et al., 2018[68]), recent studies exhibited that exosomes are involved in the diseases' pathological processes (Hamlett et al., 2018[50]; Howitt and Hill, 2016[55]; Saeedi et al., 2019[98]). In this respect, growing bodies of evidence are available suggesting that exosome quantity and/or quality may be altered in some diseases and these changes may be consequently involved in these diseases' progression (Whiteside, 2016[123]). In diseases' situations, exosomes change in two ways as follows: either their numbers change or their contents (Matsumoto et al., 2016[82]; Riva et al., 2019[95]). 

After a stroke, exosomes are synthesized and secreted by brain cells, which may evoke differential responses in different cells. It seems that exosomes have important functions in neuroprotection, angiogenesis, neurogenesis, and neurological recovery enhancing (Otero-Ortega et al., 2019[90]). In addition, the release of exosomes from the damaged central nervous cells after a stroke also has some adverse effects such as the onset and progression of neurodegenerative and neuroinflammatory diseases (Porro et al., 2015[93]), which are discussed later.

As noted earlier, following stroke, time-dependent changes are evident in gene expression. Notably, c-fos, BDNF, glial fibrillary acidic protein (GFAP), and heat shock protein 70 (HSP70) are among those genes that are affected in such situation. These changes may alter the response of non-injured brain regions, which consequently lead to secondary injury (Dietrich et al., 2000[36]). Based on the above-mentioned studies, we can postulate that exosomes might be involved in the transportation of stress proteins and neurotrophic factors after stroke.

Many evidences are also published on analyzing protein content of exosomes and it was revealed that exosomes contain some proteins involved in modulating angiogenesis and neurogenesis, suggesting that exosomes might also participate in this process (Bátiz et al., 2016[15]; Han et al., 2019[51]). Recent findings in this regard also suggest that exosomes released from neural stem/progenitor cells could promote neuronal differentiation and neurogenesis through transferring key miRNAs, and provide insights for developing novel cell-free therapeutic strategies for neurological disorders (Ma et al., 2019[80]). Furthermore, stem cell-derived exosomes have been shown to promote angiogenesis in isolated endothelial cells and murine models of vessel growth through stimulating both receptor-mediated and genetic mechanisms by directly transferring proteins, RNA or microRNA into the cytoplasm of target cells (Sahoo et al., 2011[99]).

Thus, exosomes might be involved in the transportation of the components produced after stroke, which might contribute to future brain recovery. 

### The role of exosome in post-stroke mediated promotion of neurodegenerative diseases

Recently, studies have shown that an unanticipated similarity exists between protein aggregates in chronic neurodegeneration and acute ischemic injury (Kahl et al., 2018[60]). Accordingly, this similarity showed a molecular overlap between ND and ischemic stroke. Some studies demonstrated several significant changes in the patterns of mRNA expression and protein in the blood after ischemic stroke (Dagonnier et al., 2018[31]; Zhang et al., 2018[131]).

**Figure 3:**
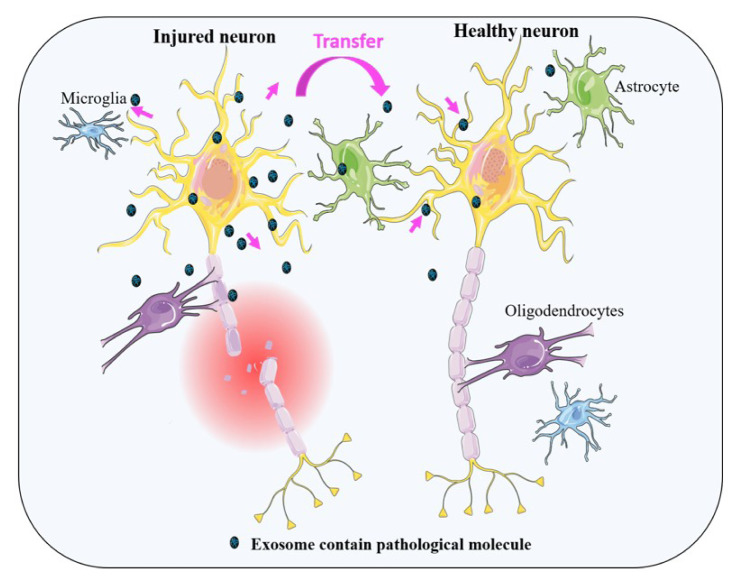
This schematic representation illustrates the release of exosomes from injured neural cells. These exosomes contain numerous types of signaling or pathological molecules which can be transferred from cell to cell. See text for complete details.

In an interesting study, Kahl et al. have shown that by passing 1 hour from reperfusion due to an acute ischemic injury and even before commence of cell death, some aggregated proteins such as TAR-DNA-binding protein 43, FUS, hnRNPA1, PSF/SFPQ, and p54/NONO, common with ND, can be formed (Kahl et al., 2018[60]). Correspondingly, one of these proteins is TDP-43, which has been observed simultaneously with tau and α-synuclein in patients with AD and dementia who had Lewy bodies (DLB) (Higashi et al., 2007[53]). On the other hand, some proofs are published showing that these proteins can be secreted via exosomes (Iguchi et al., 2016[56]; Sproviero et al., 2018[107]; Zhang et al., 2015[132]). Thus, it is possible that exosomes might be involved in the transportation of pathological proteins, which subsequently attribute to the induction of NDs after stroke (Figure 3(Fig. 3)).

As mentioned earlier, miRs are shown to participate in the post-stroke events (Nampoothiri and Rajanikant, 2019[87]). On the other hand, it has been proven that miRs are actively involved in pathological changes seen in both stroke and ND (Godlewski et al., 2019[45]). These proofs imply that miRs may be one of the factors linking these two entities.

In this respect, a number of studies have shown that plasma concentrations of miR-124 and miR-9 increase after stroke (Ji et al., 2016[58]; Laterza et al., 2009[67]; Weng et al., 2011[122]). Likewise, multiple lines of evidence indicated that miR-9 is upregulated in the serums of AD patients (Delavar et al., 2018[33]; Goh et al., 2018[46]; Tan et al., 2014[112]; Xie et al., 2017[125]). Therefore, the high plasma concentrations of miR-9 in both stroke and ND suggest that miR-9 may remain at a high level for a long time after stroke and this might be, at least in parts, responsible for the development of disorders that are emerged after stroke. Furthermore, as exosomes play a significant role in the transference of miRs including miR-9 (Baroni et al., 2016[13]; Schwarzenbach and Gahan, 2019[103]; Yang et al., 2017[127]), they mediate the distribution of miR-9 after stroke that could be considered as a possible mechanism in post-stroke promotion of ND pathogenesis. In contrast to miR-9, miR-124 significantly reduces in ND. A recent report has been published showing that the plasma concentration of miR-124 has significantly reduced in PD patients in comparison to control (Li et al., 2017[69]). Likewise, another study indicated that miR-124 expression decreased in the MPTP model of PD and its upregulation reduced the loss of dopaminergic neurons (Wang et al., 2016[120]). In the same way, there are studies indicating that miR-124 levels are gradually decreased in AD, which might have roles in the pathogenesis of AD (An et al., 2017[6]; Fang et al., 2012[42]; Yue et al., 2020[130]; Zhao et al., 2019[137]; Zhou et al., 2019[138]).

As noted previously, miR-124 was shown to be increased after stroke, but the above-mentioned studies reported that miR-124 expression decreased in ND. Therefore, the question is how these differences could be explained when we seek a relationship between stroke and ND?

One response to this question is the effect of time. In this regard, the above-mentioned studies showed the increased level of miR-124 in a short time after stroke; however, another study did not detect these changes in a longer time. Accordingly, a study performed on both patients and animal models of stroke have shown significantly elevated plasma concentrations of miR-124 at the acute injury phase. However, it was shown that it gradually decreased during the delayed recovery phase, and finally restored to normal levels at the final recovery phase (Wang et al., 2018[121]). 

Nevertheless, this study did not report plasma concentrations of miR-124 in more prolonged time points. Given the downward trend of miR-124, it is possible that concentration of miR-124 might even fall below the normal level in more prolonged intervals, but this hypothesis, which is needed to be tested in future studies, could resolve the discrepancy between these two entities. Altogether, these findings suggested that it is possible that miRs alterations might be involved in those disorders evolved after stroke. On the other hand, miRs are dispensed by exosomes; therefore, it is feasible to assume that exosomes participate in the alteration of miRs seen in pathological events, which occur after a stroke.

Generally, there was no report on the effect of exosomes and their cargos on the initiation and progression of ND after stroke, but it seems that various types of exosomes could affect the progression of ND in patients who experienced stroke.

## Concluding Markers and Future Perspectives

Some degrees of spontaneous recovery of function occur resulted from brain reorganization after stroke; however, at the same time, some changes are commencing that continue for a long time, which may result in adverse effects (Bang and Kim, 2019[10]). We can follow two strategies to reduce post-stroke complications, promote endogenous repair processes by introducing the exosome that carries benefit cargo, and prevent the spread of pathological components by inhibiting the release of exosome with pathological cargo or in an inappropriate time.

In line with the first strategy, there is evidence suggesting that exosomes obtained from stem cells mediate the beneficial effects for stroke, which may be associated with the enhanced neurogenesis, angiogenesis, and neuroprotection (Bang and Kim, 2019[10]). However, some studies indicated that the engineered exosomes derived from stem/progenitor cells containing the selected miRNA, have more potent therapeutic effects in stroke (Liu et al., 2013[75]). Moreover, in agreement with the second strategy, some studies have focused on investigating agents to block the release of subpopulations of the exosome as research tools (Catalano and O'Driscoll, 2020[23]). Therefore, exosomes can replace cell-based therapies due to their unique characteristics such as low immunogenicity and adverse effects (Sauter, 2017[101]). However, many problems with exosomes still remain unresolved.

There are multiple questions and challenges in the formation of an exosome by its release from the brain after stroke and exosome therapy. Understanding how exosomal cargo is affected by stroke and how these components are loaded into exosomes and reach particular cell types targeted by this exosome, provide more details for the mechanism of ND after stroke in order to prevent it. In this study, we proposed that some pathological components, such as proteins and miRs that are common between stroke and ND and transported by the exosome, are involved in their association. Although long-term studies can better address the relationship between these complex pathological components in the above-mentioned disorders, this novel approach may eventually lead to a novel target for the treatment of stroke and neurological diseases.

## Notes

Mahmoudreza Hadjighassem and Rasoul Ghasemi (Department of Physiology, School of Medicine, Shahid Beheshti University of Medical Sciences, Tehran, Iran and Neurophysiology Research Center, Shahid Beheshti University of Medical Sciences, Chamran highway, Velenjak, Tehran, Iran; Phone: + 98 21 22439971, E-mail: Rghasemi60@sbmu.ac.ir, r_ghasemi60@yahoo.com) contributed equally as corresponding authors.

## Acknowledgement

We gratefully thank the Neurophysiology Research Center, Shahid Beheshti University of Medical Sciences for supporting this work.

## Conflict of interests

The authors declared no conflict of interest.
